# Pelvic Actinomycosis Associated with an Intrauterine Contraceptive Device Demonstrated on F-18 FDG PET/CT

**DOI:** 10.3390/diagnostics5030369

**Published:** 2015-08-28

**Authors:** Danijela Dejanović, Jan Anders Ahnlide, Cecilia Nilsson, Anne Kiil Berthelsen, Annika Loft

**Affiliations:** 1 Department of Clinical Physiology, Nuclear Medicine & PET, Rigshospitalet, University of Copenhagen, 1165 Copenhagen, Denmark; E-Mails: j.a.ahnlide@telia.com (J.A.A.); anne.kiil.berthelsen@regionh.dk (A.K.B.); annika.loft.jakobsen@regionh.dk (A.L.); 2 Juliane Marie Centre for Children, Women and Reproduction (JMC) Rigshospitalet, Copenhagen University Hospital, 1165 Copenhagen, Denmark, Denmark; E-Mail: cecnilsson@gmail.com

**Keywords:** FDG, PET/CT, intrauterine device, actinomycosis, frozen pelvis

## Abstract

A 44-year-old woman with a history of dysmenorrhea, obstipation, and low back pain was investigated for gynecological disorder. Physical examination indicated a “frozen pelvis”. Ultrasound examination revealed the ovaries adherent to the uterus, bilateral ovarian cysts, and an intrauterine contraceptive device *in situ*, which reportedly had been in place for 19 years. Prior to a scheduled laparoscopy, the patient returned with oedema of the lower abdomen and legs, fatigue, and weight loss. Laboratory findings included elevated CA-125, anemia, leucocytosis and high C-reactive protein. Pelvic actinomycosis was subsequently diagnosed. We report the PET/CT appearance of this condition.

**Figure 1:**
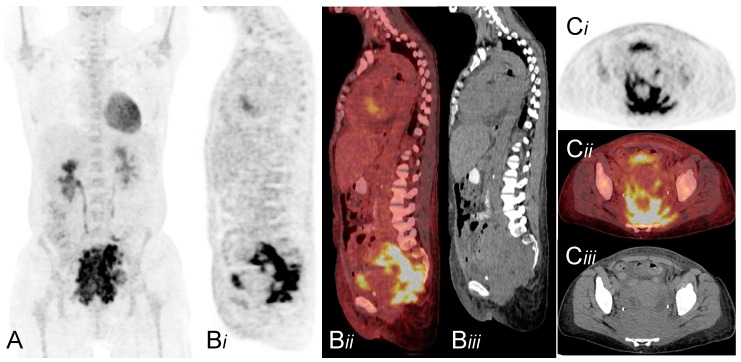
A 44-year old woman with several months’ history of dysmenorrhea, obstipation, and low back pain was examined. The patient’s history and ultrasonographic findings, with the ovaries adherent to the uterus and possible endometriomas, were suggestive of endometriosis. Additionally, an intrauterine device (IUD) was in place, which had not been replaced in 19 years. Six weeks after the initial consultation, the patient returned with oedema of the lower abdomen and legs, weight loss, and fatigue. The IUD had been removed. Laboratory findings showed erythropenia, leucocytosis, elevated C-reactive protein, and creatinine. Renography showed bilateral urinary tract obstruction and JJ-catheters were implanted. An F-18 fluoro-deoxyglucose (FDG) PET/CT scan with oral but no intravenous contrast, primarily planned to rule out ovarian carcinoma, was performed. PET-CT (**A** Maximum intensity projection. **B*i*-*iii*** Sagittal projection; PET, fused and CT scan respectively. **C*i*-*iii*** Transverse projection; PET, fused and CT scan respectively) revealed a diffusely infiltrative, FDG-avid (SUVmax 18, 0), pelvic mass mainly located in the pre-sacral region extending around the vagina, the intestines and through the greater sciatic foramen intermuscularly on the left side (**C*i***, **C*ii***, arrow). Subcutaneous oedema of the lower part of the body is seen on CT (**B*iii***, **C*iii***). The red bone marrow is seen with diffuse high uptake consistent with anemia and infection (**A**). Based on PET/CT findings alone, no definite diagnosis was possible and malignancy could not be ruled out. Furthermore, the interpretation on the CT scan was compromised due to the absence of intravenous contrast. Actinomycosis was diagnosed by endometrial histology. The patient was treated successfully with high dose intravenous benzylpenicillin and no surgery was required. *Actinomyces* species are slow-growing, Gram-positive, anaerobic bacteria [1]. Pelvic actinomycosis infection is rare and often associated with IUD in women [2,3,4]. Actinomycosis yields a characteristic granulomatous response with abscess formation, necrosis, and reactive fibrosis with a sub-acute-to-chronic course [3,4,5,6]. Advanced pelvic actinomycosis with formation of solid masses can be difficult to differentiate from pelvic neoplasia, such as ovarian carcinoma, as it can compress nearby structures, as well as invade surrounding tissue [3,5,7,8]. The disease has multiple clinical presentations and non-specific CT findings [4,9] and can lead to extensive surgery before correctly diagnosed [6]. As demonstrated by our F-18 FDG PET/CT scan and previously described cases [4,9], pelvic actinomycosis is an important differential diagnosis in imaging when presented with an infiltrative mass with a tendency to invade across tissue boundaries. This is especially true in women with a history of IUD and signs of infection.
